# Editorial: Novel Treatment Strategies for Myeloproliferative Neoplasms

**DOI:** 10.3389/fonc.2021.762928

**Published:** 2021-09-15

**Authors:** Ken-Hong Lim, Shinobu Matsuura, Bing Xu

**Affiliations:** ^1^Division of Hematology and Oncology, Department of Internal Medicine, MacKay Memorial Hospital, Taipei City, Taiwan; ^2^Department of Medicine, MacKay Medical College, New Taipei City, Taiwan; ^3^Department of Medicine–Whitaker Cardiovascular Institute, Boston University School of Medicine, Boston, MA, United States; ^4^Department of Hematology, The First Affiliated Hospital of Xiamen University and Institute of Hematology, School of Medicine, Xiamen University, Xiamen, China; ^5^Key Laboratory of Xiamen for Diagnosis and Treatment of Hematological Malignancy, Xiamen, China

**Keywords:** myeloproliferative neoplasms, chronic myeloid leukemia, CALR, MPL, tyrosine kinase inhibitor

The myeloproliferative neoplasms (MPNs) are a group of clonal stem cell diseases with heterogeneous clinical features and laboratory characteristics. Among the classic MPNs, chronic myeloid leukemia (CML) is characterized by the presence of Philadelphia chromosome or the *BCR*-*ABL1* fusion transcript (1). The Philadelphia chromosome-negative classic MPNs include polycythemia vera (PV), essential thrombocythemia (ET) and primary myelofibrosis (PMF). In the majority of patients with PV, ET and PMF, driver mutations including *JAK2*, *MPL* or *CALR* mutations can be detected, and all these driver mutations result in the activation of the JAK-STAT signaling pathway (2–7). The aberrant activation of tyrosine kinase signaling pathway in MPNs has become attractive target for therapeutic intervention and has lead to the innovation of targeted therapy in MPNs during the past 3 decades. The development of selective BCR-ABL1 tyrosine kinase inhibitors (TKIs) has dramatically improved the prognosis for patients with CML, and over 80% of patients can now become long-term survivors under the treatment of BCR-ABL1 kinase inhibitors (8). Following the highly successful targeted therapy in CML, selective JAK inhibitors have also revolutionized the treatment and outcome of Philadelphia chromosome-negative classic MPNs especially PMF and PV (2, 3). However, the management of patients with MPNs is still challenging. In this Research Topic, we discuss some novel strategies for the treatment and management of MPNs including minimizing the risk of thrombotic events, novel approaches targeting CALR and MPL, and the goal of long-term treatment-free remission (TFR) in CML.

Both arterial and venous thrombotic events are the largest contributor to morbidity and mortality in PV and ET (9). In their review, Tremblay et al. describe the burden of thrombosis in PV and ET and explored their pathophysiologic mechanisms including hematologic parameters, mutated *JAK2* and the proinflammatory milieu. They point out that age and prior thrombosis remain to be potent predictors of subsequent thrombosis both in PV and ET. They also assess and summarize the evidence behind currently available therapies for thrombosis in PV and ET. In order to accurately assess reduction of thrombotic events, they propose that surrogate endpoint validation and biomarker development for thrombotic events should be incorporate into clinical trials in PV and ET.

Cattaneo et al. focus on the clinical, pathological and molecular features of triple-negative ET. They reviewed the bone marrow histology and determined that most of the cases displayed a classical ET morphology with several unique characteristics in megakaryocyte morphology. In their study, next-generation sequencing was used to determine the molecular profiles of 40 triple-negative ET patients. Nucleotide variants were detected in 30 patients (87.5%), and mostly involved the *TET2* and *KIT* genes suggesting that a sizeable proportion of triple-negative ET patients do have a clonal disease.

The *CALR* exon 9 and *MPL* exon 10 mutations are identified in ~25% and ~5% of patients with Philadelphia chromosome-negative MPNs, respectively (2, 3). Because The *CALR* exon 9 indel mutations generate a novel mutant C-terminus with immunogenicity, Grauslund et al. develop a therapeutic cancer vaccine with the peptide CALRLong36 against mutant CALR as a new treatment modality in CALR-mutant MPN, and tested its safety and efficacy in ten *CALR*-mutated MPN patients in a phase I clinical trial. Their results show that the peptide CALRLong36 vaccine is safe and tolerable although the vaccines did not induce any clinical responses. It is noteworthy that the majority of MPN patients displayed a marked T-cell response to the vaccine at the end of the trial. They suggest that vaccines directed against mutant CALR may be used with other cancer therapeutic modalities to enhance the anti-tumor immune response.

In their review, Spivak et al. summarize the role of MPL and thrombopoietin (THPO) in MPN hematopoietic stem cell (HSC) pathophysiology and propose a hypothesis that MPL offers a selective therapeutic target in MPNs. They suggest that the unique dependence of HSC on the MPL–THPO axis and the unusual pathophysiology of MPL in the MPNs have generated a therapeutic target of opportunity to suppress MPN HSC while sparing normal HSC. They have utilized a *JAK2* V617F transgenic mouse model to confirm their hypothesis. They found that the PV histologic phenotype of the *JAK2* V617F transgenic mouse model was abolished and the marrow HSC markedly decreased after crossing with an MPL knockout mouse model. Therefore, MPL plays an essential role in this *JAK2* V617F transgenic mouse model of PV. Finally, they discuss the control of THPO production as a novel therapeutic approach for MPNs through gene silencing of hepatic targets. To achieve this therapeutic goal of lowering THPO levels and platelet counts, a technology using the Ashwell–Morrell receptor as entry for modified RNA inference into hepatocytes is proposed.

Selective BCR-ABL1 TKIs are cornerstone in the management of CML (10, 11). In CML patients achieving durable deep molecular response, long-term TFR becomes an important treatment goal with the hope for a cure in these patients. Mu et al. review several novel and potentially curative combination treatment strategies targeting leukemic stem cells in CML. Interferon-α, BCL-2 inhibitor venetoclax, JAK2 inhibitor ruxolitinib, the peroxisome proliferator-activated receptor gamma agonist pioglitazone and the ABL myristoyl pocket inhibitor asciminib have been used in combination with various TKIs in CML. Promising results are seen in many of these novel combination therapies and clinical trials are ongoing with the results eagerly awaited in the near future. Furthermore, immunological strategies that are being tested in clinical trials for the treatment of CML include the therapeutic effect of BCR-ABL1 immunopeptides, leukemia associated antigens, and immune checkpoint blockade. With all these novel treatment strategies, it is anticipated that the outcome of CML will be continuously improved and long-term TFR may be achieved in the majority of patients.

In conclusion, the collection of original studies and review articles in this Research Topic provide important insight and progress in the treatment for MPNs. Advancement in this field will rely on the study of clinical and molecular characteristics of patients, translation research to look for potential novel treatment targets, and clinical trials testing new combination treatments.

## Author Contributions

K-HL wrote the draft manuscript. All authors contributed to the article and approved the submitted version.

## Funding

K-HL was supported by the grants sponsored by the Ministry of Science and Technology of Taiwan: MOST 107-2314-B-195-011-MY3 and 110-2314-B-195-008. The funder has no role in the preparation or decision to publish of the manuscript.

## Conflict of Interest

The authors declare that the research was conducted in the absence of any commercial or financial relationships that could be construed as a potential conflict of interest.

## Publisher’s Note

All claims expressed in this article are solely those of the authors and do not necessarily represent those of their affiliated organizations, or those of the publisher, the editors and the reviewers. Any product that may be evaluated in this article, or claim that may be made by its manufacturer, is not guaranteed or endorsed by the publisher.
